# Predicting developmental norms from baseline cortical thickness in longitudinal studies

**DOI:** 10.1186/s13293-026-00846-4

**Published:** 2026-02-10

**Authors:** Philipp Seidel, Tobias Kaufmann, Thomas Wolfers

**Affiliations:** 1 Department of Psychiatry and Psychotherapy, Tübingen Center for Mental Health, University of Tübingen, Tübingen, Germany; 2 German Center for Mental Health (DZPG), partner site Tübingen, Tübingen, Germany; 3 Centre for Precision Psychiatry, University of Oslo, Oslo, Norway; 4 Institute for Psychology, Friedrich Schiller University Jena, Jena, Germany

## Abstract

**Summary:**

Normative modeling has been applied to study how brain measures, such as gray matter thickness or volume, change across development. These models help identify how an individual’s brain may differ from what is typical for their age or sex, which could eventually support more personalized treatments. However, most existing models use only one-time (cross-sectional) data, meaning they cannot capture how the brain changes over time. Longitudinal data, tracking the same individuals across multiple time points, is more informative but harder and more expensive to collect. We analyzed brain scans from over 6000 young people in the Adolescent Brain Cognitive Development (ABCD) study, about half of whom were girls. Each participant had brain scans at the start of the study, two and four years later. We deployed Baseline-Conditioned Norms (B-Norms) that used cortical thickness derived from each person’s first scan and their ages at baseline and follow-up timepoint to predict cortical thickness at follow-up. We compared this to Cross-Sectional Norms (C-Norm), which only used age to predict thickness at follow-up. As expected, B-Norms predicted cortical thickness more accurately. Importantly, they were also better at detecting brain differences linked to puberty, especially in girls. Our findings suggest that our here proposed B-Norms may capture more developmental variance and may be more sensitive to sex-specific brain development over time during puberty. Therefore, B-norms may constitute a valuable complement to established C-norms.

**Background:**

Normative models have gained popularity in computational psychiatry for studying individual-level differences relative to population norms in biological data such as brain imaging, where measures like cortical thickness are typically predicted from variables such as age and sex. Nearly all published models to date are based on cross-sectional data, limiting their ability to predict longitudinal change.

**Methods:**

Here, we used longitudinal brain data from the Adolescent Brain Cognitive Development (ABCD) study, comprising cortical thickness measures from 180 regions per hemisphere in youths at baseline (*N* = 6179; 47% females), 2-year (*N* = 6179; 47% females), and 4-year (*N* = 805; 45% females) follow-up. A training set was established from baseline and 2-year follow-up data (*N* = 5374; 47% females), while data from individuals with all three time points available served as an independent test set (*N* = 805; 45% females). We developed sex-specific Baseline-Conditioned Norms (B-Norms) that predict brain region thickness at follow-up based on baseline thickness, baseline age, and follow-up age, and compared them to sex-specific Cross-Sectional Norms (C-Norms) that predict thickness at follow-up based on age alone.

**Results:**

As expected, out-of-sample testing in 2-year and 4-year follow-up data showed that B-Norms consistently provided better fits than C-Norms for nearly all cortical regions. Explained variance was higher in B-Norms than in C-Norms. No significant differences between time points (*p* = 0.45) were detected. Repeated measures ANOVA revealed differences in higher-order moments (e.g., skewness and kurtosis) for both models; for example, skewness varied by model, sex, time point, and their interactions. We showed that four regions were associated with pubertal changes in B-Norms but not in C-Norms, suggesting enhanced sensitivity of B-Norms to developmental processes.

**Conclusion:**

Together, our findings highlight the potential of B-Norms for capturing normative variation in longitudinal structural brain change, suggesting that they may constitute a valuable complement to existing C-Norms.

**Supplementary Information:**

The online version contains supplementary material available at 10.1186/s13293-026-00846-4.

## Highlights

- Baseline-Conditioned (B-Norm) models performed very well in predicting cortical thickness at follow-up.
- B-Norm deviation scores show stronger associations with pubertal progression in females than C-Norm deviations, indicating higher developmental sensitivity.
- B-Norms may therefore complement existing norms with norms sensitive to longitudinal change.

**Supplementary Information:**

The online version contains supplementary material available at 10.1186/s13293-026-00846-4.

## Background

The predominant analytical approach in neuroimaging for identifying disease-related brain alterations has been case-control comparisons. Over the past decade, this group-level approach has increasingly been complemented by normative modelling - a framework that enables the quantification of individual deviations from expected neurobiological patterns relative to a reference population [1, 2]. In brief, normative models estimate centiles of variation across neurobiological or behavioral measures, allowing inferences about an atypical development at the individual level without the need for predefined diagnostic groups.

A key strength of normative modelling lies in its flexibility to model diverse mappings across different phenotypic domains, ranging from neuroimaging measures (e.g., cortical thickness/volume) to behavioral or demographic traits [3]. Therefore, this modeling approach is particularly well-suited for lifespan research, where inter-individual variability often reflects subtle and complex developmental or degenerative processes [3–6]. For example, deviations from normative neurodevelopmental trajectories have been implicated in the pathogenesis of psychiatric and neurodevelopmental conditions [7]. More recent applications have used normative models to investigate cognitive decline and deterioration or increases of structural brain measures (e.g., cortical thickness/volume) in aging and neurodegenerative disorders [4, 8–13]. These models further provided valuable insights into heterogeneity in psychiatric conditions such as attention-deficit/hyperactivity disorder [14, 15], schizophrenia [14, 16–18], autism spectrum disorder [16, 19–22], and Alzheimer’s/dementia [23–27].

Despite these advances, most normative modelling studies have relied on cross-sectional data. To some extent, this limits their capacity to accurately predict within-subject longitudinal change. A recent study showed evidence that cross-sectional models may underestimate dynamic brain changes over time [8, 28, 29]. Although recent studies have begun to incorporate and investigate longitudinal data (e.g., [30]), some of which, however, still apply cross-sectional models to individual data points, rather than leveraging longitudinal information [27, 31, 32]. Among other disciplines developmental neuroscience has emphasized the need for longitudinal designs and investigations to capture changes during brain maturation - a crucial developmental period for which mounting evidence of vulnerability to psychiatric illness has been put forth [33–37]. Recent findings suggest that modelling deviations in the timing of pubertal onset - such as early puberty onset - can enhance the prediction of later mental health outcomes [34, 38]. Such evidence highlights the translational potential of (longitudinal) normative models for early detection and thus possible interventions.

To this end, we deployed Baseline-Conditioned Norms (B-Norms) that use baseline cortical thickness data and age to predict cortical thickness at later timepoints. We compared them to classical Cross-sectional Norms (C-Norms) where cortical thickness at a given timepoint is predicted using the age at that timepoint alone. Specifically, we used data from the Adolescent Brain Cognitive Development (ABCD) Study [39], comprising baseline data from 5374 participants (2515 female; baseline: mean ± SD age = 118.78 ± 7.44 months), and data at 2-year follow-up which were used to train our models. Furthermore, we used data from participants who participated at three timepoints to test our models: 805 participants (366 female; baseline: mean ± SD age = 119.28 ± 7.34; 2-year: mean ± SD age = 143.22 ± 7.49; 4-year: mean ± SD age = 168.45 ± 7.77). Cortical thickness measurements were derived from 360 regions of interest (ROIs) based on the Glasser atlas (Glasser, 2016). For both B- and C-Norm models, we trained them for each brain region separately, hypothesizing that B-Norm models utilizing longitudinal data would show enhanced sensitivity to developmental changes within the respective developmental time period. To validate this, we examined the relationship between model-derived deviation scores and pubertal development as determined by the Pubertal Development Scale (PDS) scores. Validation was performed in the held-out test set. PDS scores acquired at 2-year and 4-year follow-up were statistically associated with the respective model-derived deviation scores of C- and B-Norm models. Our findings aim to contribute to the ongoing refinement of normative modelling approaches, especially in the context of individual-level longitudinal developmental predictions.

## Methods

### Dataset

We made use of longitudinally acquired data of the Adolescent Brain Cognitive Development (ABCD, Casey et al. [39]) study. In the ABCD dataset children are recruited at ages 9 to 10 with the aim of characterizing brain developmental trajectories. To this date more than 11.868 children were recruited across 21 different sites in the United States of America. Study procedures have been approved by either the local site Institutional Review Board (IRB) or by local IRB reliance agreements with the central IRB at the University of California. All participants and their parents or legal caregivers provided written informed consent. Data for the current study was obtained from ABCD release 5.1 utilizing phenotypic and imaging data from the baseline, 2-year, and 4-year follow-up visits.

### Data selection and preprocessing

**Demographics.** The ABCD project provides a multitude of tabulated data. Here, we made use of the following files: *abcd_p_demo* to extract sex and ethnicity; *abcd_y_lt* to extract the interview age at baseline and follow-up visits; the *ph_y_anthro* file to compute body mass index (BMI) which we use to exclude participants with unusually large BMI; and *mri_y_adm_info* for information about scan sites which we use as covariates during model training and prediction. We additionally computed mean puberty score (PDS) as rated by the youths’ caregivers from data of the *ph_p_pds* file to associate deviation scores with youth’s pubertal progress.

**Puberty scores.** We calculated a summary statistic representing progress in pubertal development using items of the Pubertal Development Scale (PDS; Herting et al. [40]; Kraft et al. [41]) at each study visit. This rating scale was designed to reflect Tanner stages without the need for physical examination [42, 43]. In this questionnaire, a child’s pubertal development is assessed using a four-point Likert scale ranging from ‘has not begun’ to ‘completed’. These items were specific to certain physical characteristics (including skin changes, breast development, deepening in voice, etc.). Please note that some items were administered based on sex. For example, the onset of menarche was exclusively asked for females and is a binary (i.e., either 1 or 4) rating. The ratings are either provided by the children or their caregivers. In this study, we focus on ratings provided by the caregivers for two reasons: (a) the self-reported ratings appeared less reliable and (b) more data is available for caregiver ratings [42].

**Cortical thickness data.** We preprocessed the raw structural data of the ABCD on an in-house cluster computer (Ubuntu 22.04) using the recon-all functionality of Freesurfer v7.4.1 [44]. Cortical thickness values were calculated and extracted for 180 regions per hemisphere as defined by the Glasser atlas [45]. In addition, we stored Euler numbers which we used to exclude badly reconstructed data during preprocessing.

**Fig. 1:**
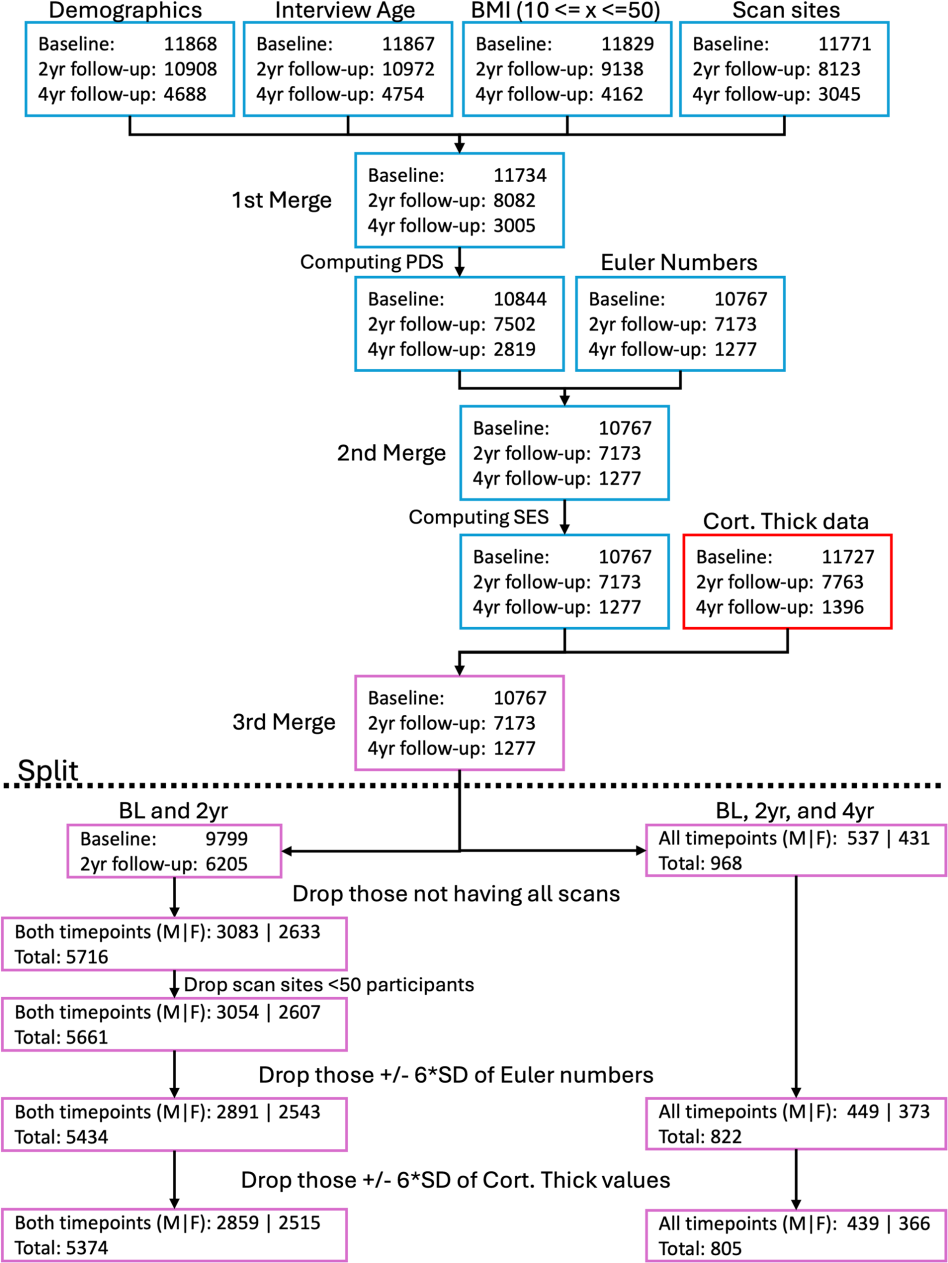
Data preprocessing flowchart of the ABCD dataset. We first loaded data from the ABCD study. Namely demographics (abcd_p_demo), interview age (abcd_y_lt), we computed BMI (ph_y_anthro) and excluded all participants that had a BMI between 10 and 50 and loaded the scan site information (mri_y_adm_info). We then merged these files (1st Merge). After that, we computed PDS scores (ph_p_pds) as described previously [40, 41]. The resulting data was then merged with Euler Numbers (obtained from inhouse Freesurfer reconstruction; 2nd Merge). After that we computed socioeconomic status (abcd_p_demo) and merged the resulting data with the cortical thickness data we received from our inhouse Freesurfer preprocessing. This resulted in the data represented by “3rd Merge”. We then retained data for all participants who had Baseline, 2 year-, and 4-year follow-up data (right path below the dashed “Split” line). For the remaining data (left path below the “Split” line), we first retained only those participants who had both Baseline and 2 year follow-up data. For both paths, we then removed those participants whose Euler Numbers exceeded 6*SD from the mean. Lastly, we removed all those participants who had unusually large (again exceeding 6*SDs from the mean) cortical thickness values within any Glasser brain region.

**Preprocessing.** Before preprocessing, data comprised of 11,868 participants with a baseline measure, 10,908 participants with 2-year follow-up data, and 4688 participants with 4-year follow-up data. We excluded all those participants which had only a single measurement. Additionally, we excluded all participants with BMIs of less than 10 or larger than 50. We included this step because BMI has previously been associated with changes in cortical thickness in adolescents [46] as well as in adults [47, 48]. Furthermore, we excluded all those participants who had no valid or no pubertal development score (PDS) rating and cortical thickness measures available. We then separated those participants with only baseline- and 2-year follow-up data into a subset and those with baseline-, 2-year, and 4-year data into another. For both subsets we used the Euler numbers and computed their mean and standard deviation for each of the ABCDs scan-sites. We then excluded all participants whose Euler numbers were larger than 6 SDs from the mean for a given scan-site. This was to ensure that no extremely badly reconstructed data was used (for overview plots see Supplementary Figs. S1 and S2). Lastly, we performed a similar exclusion step for cortical thickness values. We again calculated the mean cortical thickness and standard deviation across subjects for each ROI and excluded all participants who exceeded the 6 SDs threshold. This was done to exclude extreme outliers in cortical thickness, likely originating from bad Freesurfer reconstructions. This is important as normative curves are calculated by using the minimum and maximum values of the training data and such extreme values would distort the trajectories. For details on the stepwise number of excluded participants see Fig. 1 below.

**Train test and splits.** After preprocessing we designated those participants with only baseline (female: *N* = 2515, age = 118.30±7.39 [mean±SD]; male: *N* = 2859, age = 119.06±7.49) and 2-year follow-up measurements (female: age = 142.92±7.81; male: age = 143.74±7.81) as the training dataset. Participants with baseline (female: *N* = 366, age = 118.87±7.47; male: *N* = 439, age = 119.62±7.41), 2-year follow-up (female: age = 142.73±7.31; male: age = 143.64±7.62), and 4-year follow-up (female: age = 167.82±7.48; male: age = 168.98±7.99) data were used for testing. This approach ensured independent samples during the training process as well as for testing. The splits did not differ significantly on core demographic variables such as age or BMI at baseline but showed some statistical differences as determined by a Welch’s t-test in PDS scores. We argue that this difference, while unfortunate, is barely avoidable as stratifying for these variables would have resulted in a much smaller test set. For more details see supplementary figs. S3−5 and the corresponding text.

### Normative modelling with bayesian linear regression

We employed normative modeling using Python 3.12.9 and the PCNtoolkit [49] package (version 0.33). Bayesian Linear Regression (BLR) with likelihood warping [50] was used to predict the target measure cortical thickness (CT) at a given timepoint from a covariance matrix including “age_bl_ and site” for the classic Cross-Sectional normative models (C-Norms) and “cortical thickness_bl_, age_bl_, age_follow−up_, and site” for the Baseline-Conditioned normative models (B-Norms). Input age values were the age in months. Sinarcsinsh was employed for warping [51]. Site covariates were encoded as one-hot vectors (i.e., one vector per site where the respective column was set to “1” for a particular site). For each of the 180 brain regions for both hemispheres as defined by the Glasser atlas [45], cortical thickness is predicted as:

$$\:{CT}_{bl}={age}_{bl}+site\_covariates$$

for the C-Norms, and

$$\begin{aligned}\:{CT}_{2yr}&={CT}_{bl}+{age}_{bl}+{age}_{follow-up}\\ &\quad +\:site\_covariates\end{aligned}$$

for the B-Norms. Where the subscript ‘*follow-up*’ in the second age-parameter corresponds to either the age at the 2-year or 4-year follow-up visit. While the age variables certainly are correlated to one other, we argue using them this way is not of a particular issue as we are not interpreting the contribution of individual variables.

Both models were optimized using the powell algorithm, and results are based on models trained on the training split and evaluated on the independent test set. We assessed the model fit for each brain region using several metrics, including Pearson’s correlation between observed and predicted measures, root-mean-squared error (RMSE), standardized mean-squared error (SMSE), explained variance (EV), and mean squared log-loss (MSLL). Additionally, we evaluated skewness and kurtosis to estimate higher-order moments beyond the mean in the test set [52].

### Comparing performance measures

We used the ANOVA function from pingouin python package to perform 2-factor repeated-measures analysis of variance (rm-ANOVA) to statistically assess differences in performance metrics of the C- and B-Norms separately for both males and females. The dependent variables were the performances measures as defined earlier. Our within-subject factors were MODEL (levels: C-Norm and B-Norm) and TIMEPOINT (levels: 2-year and 4-year follow-up), the identifier variable were the regions of interest as defined by the Glasser atlas. We show statistics for explained variance, skewness, and kurtosis in the main text. Mean squared log loss (MSLL), (standardized) mean squared error ([S]MSE), root-mean squared error (RMSE), and Bayesian Information Criteria (BIC) are presented in the supplementary material.

### Validations of normative models against puberty scores

We performed an association analysis to reveal relationships between puberty scores as rated by the youths’ caregivers and the deviation scores (i.e., z-scores) obtained from the normative models. We conducted this analysis performing generalized linear models (GLMs) with a Gaussian family and identity link using the statsmodels Python-package, specifically the glm function. For each sex-specific model and each ROI, we computed the association $\:{PDS}_{t}\sim{zROI}_{t}$, where $\:PDS$ are vectorized timepoint specific mean PDS scores per participant (calculated as the average across the individual Pubertal Development Scale items) and $\:zROI$ is a vector containing subjects’ deviation scores as obtained by the C- or B-Norms at follow-up time point *t* (i.e., 2-year or 4-year). This resulted in 360 z-statistics and p-values (which we corrected for multiple comparisons using the Benjamini-Hochberg [53] false discovery method), one for each ROI, for each model, and sex. Model degrees of freedom (df) were 1 and residual df differed between sexes (437 for males and 364 for females). Deviance and scale are reported in the results section. Statistics were then projected onto a surface brain and thresholded according to the critical BH values. As a supplementary analysis, we included BMI and socioeconomic status as covariates. These results are available in the supplementary materials. Furthermore, we provide the results in tabular format as supplements.

### Validation of normative models using puberty subgroups

To examine differences in positive and negative deviations across ROIs, participants were grouped into five pubertal stages (pre-, early-, mid-, late-, and post-pubertal), following Herting et al. and Kraft et al. [40, 41]. Glasser ROIs were aggregated into six lobe-level regions: occipital, frontal, temporal, parietal, insular, and cingulate – based on previously proposed definitions [54]. For each participant and lobe, we counted the number of regions exceeding z-scores of +/- 1.96, producing lobe-specific deviation vectors. These count vectors were stratified by pubertal stage, and Kruskal-Wallis tests were conducted across stages for each lobe, deviation type (positive/negative), model (cross-sectional/Baseline-Conditioned), and timepoint (2- and 4-year follow-ups), totaling 192 tests.

### Association of stable, positive or negative percentile shifts with puberty progression

We additionally validated the normative models by categorizing participants into three groups based on percentile shifts between the 2-year and 4-year follow-up data. Specifically, we defined three groups: negative (zDiff < −1), stable (−1 < zDiff < 1), and positive (zDiff > 1), with zDiff representing the change in ROI-specific z-scores over time. For each ROI, participants were assigned to one of these groups; the number of participants per group therefore varies. For each ROI a Kruskal-Wallis test was conducted to assess group differences in delta PDS scores (4-year minus 2-year). Please note: the results of this analysis are available in Sect. Validation of normative models using percentile shifts of the supplements.

## Results

### Integrating baseline thickness measures as predictors for norms in longitudinal designs

While C-Norms predict cortical thickness based on age, we here examined if a different modelling approach by using cortical thickness at baseline alongside age at baseline and follow-up as features yields more accurate predictions (B-Norms), and if the resulting deviation scores yield stronger and potentially more meaningful associations with external variables that are sensitive to change, such as pubertal development. We trained B-Norm and C-Norm models in the same training data set, separately for males and females to account for potential sex specific trajectories. To validate the trained models, we compared explained variance for our models when applied to the 2-year and 4-year follow-up held-out test data. This allowed us to assess the accuracy of fits (i.e., the center of the distribution). Additionally, we assessed differences in higher order moments (i.e., skewness and kurtosis) of the resulting deviations (i.e., z-scores) to investigate the shape and calibration of the centiles of deviation. The latter is important as it ensures that the models accurately fit to the overall distribution and thereby allow for reliable inferences [55]. While the comparison between the two models is not of most importance for this article, we include it here to allow for contextualization of the B-Norm fits with established C-Norm fits.

**Fig. 2:**
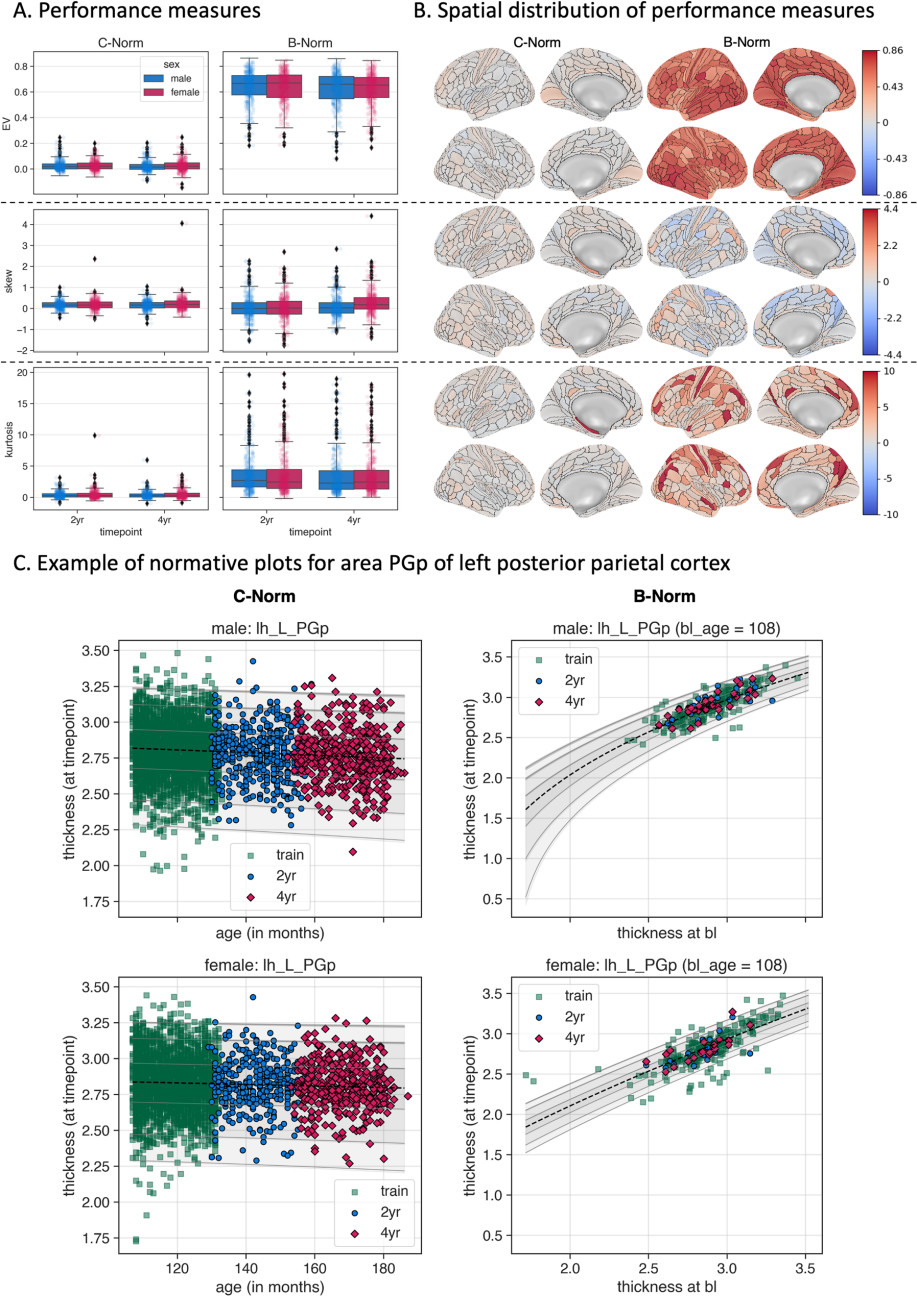
Cross Sectional Norms (C-Norm)- vs. Baseline-Conditioned Norms (B-Norm). Panel (A) depicts three performance measures of the different normative models: explained variance (top row), skew (middle row), and kurtosis (bottom row) for both models (columns). In panel (B) the first two columns correspond to the lateral and medial views of the C-Norms, the last two to the B-Norms. Warmer colors indicate higher explained variance, positive skewness or kurtosis; colder colors indicate negative skewness or kurtosis. Panel (C) shows normative plots of an example ROI (here the PGp, as defined by the Glasser atlas). The columns represent normative trajectories for the standard C-Norms (left) and the B-Norms (right). Rows correspond to sex. Within each graph, green squares indicate the training data. The black dashed lines indicate the median. The blue circles and red diamonds correspond to the 2-year and 4-year follow-up data, respectively. Grey lines indicate the centiles (1%, 5%, 25%, 75%, 95%, and 99%) and gray patches around these lines indicate the respective uncertainty. Note: given the nature of the B-Norms, we needed to plot one normative plot per baseline age. Only one baseline age (108 months) is depicted here. Others are available in the supplementary material (see Fig. S23).

Given that we used baseline cortical thickness as a feature in our B-Norm models, we expected to find better model fits when predicting thickness at follow-up for B-Norms than C-Norms. Indeed, the marked differences in the mean fit showed higher explained variance for both 2-year and 4-year follow-up data in B-Norms compared to C-Norms (Fig. 2A). The overall pattern was similar across sexes, with higher variance explained for B-Norms (mean±SD across timepoints, males: 0.635±0.125; females: 0.633±0.123) compared to C-Norms (males: 0.023±0.038; females: 0.026±0.04). On top of these differences, we observed a small model-by-timepoint interaction, indicating that the accuracy of mean fits decreased from 2-year to 4-year follow up, with largest effects in B-Norms and in males (model-by-timepoint interaction, males: F_1,359_=25.86, *p* < 5.91e^− 7^, $\eta^{2}$=0.0672; females: F_1,359_=26.04, *p* < 5.43e^− 7^, $\eta^{2}$=0.0676; post-hoc t-tests males: *C-Norm* mean-diff = 0.0038, t = 3.268, *p* < 1.18e^− 3^, *B-Norm* mean-diff = 0.0154, t = 7.016, *p* < 1.14e^− 11^; post-hoc tests in females: *C-Norm* mean-diff = 0.0006, t = 0.613, *p* < 0.54; *B-Norm*: mean-diff = 0.0129, t = 5.618, *p* < 3.88e^− 8^).

For the shape of the distribution and the centiles of the predicted z-scores, we investigated skewness and excess kurtosis (i.e., kurtosis values below or greater than 0), where values close to zero would indicate a standard normal distribution. On average, we found that skewness was closer to zero for B-Norms than C-Norms, however, variance was larger in B-Norms. We observed larger excess kurtosis for B-Norms as compared to C-Norms. The subpanels in Fig. 2A depict the distributions and the corresponding statistics for explained variance, skewness, and kurtosis, among other statistics, from our repeated measures ANOVA are detailed in the Supplement (see Figs. S9-22 and Tables S1-8). Figure 2B illustrates the spatial distribution of the performance metrics, exemplarily for females at 2-year follow-up. In the depicted female B-Norms, we found highest explained variance in large parts of the occipital (left/right PIT, VMV1, and left MT) and temporal regions (right PHT, PH, TP0J2 and left TP0J1, TE1m and STSda), and lowest explained variance scores in frontal (left/right pOFC, right OFC, 13l, and 25 and left 6d) and insular (left AAIC and PoI2, and left/right FOP3) areas. Similar results were found for the female 4-year follow-up data. Results for male B-Norms, were mostly consistent with females (see supplementary Figs. S24 and 25). Interestingly male C-Norms showed different highest explained variance scores in parietal and frontal areas for the 2-year and 4-year follow-up data. Furthermore, we saw differences between lowest explained variances in male C-Norms for the 2-year and 4-year follow-up data. Specifically, we found different areas of the frontal lobe that showed explained variances below 0 (i.e., the model performs worse than chance) for the 2- and 4-year follow-up data as well as some temporal regions in the 2-year and parietal regions in the 4-year follow-up data. These results were inconsistent with the predictions made by female C-Norms on 2-year follow-up data with largest explained variance in frontal and temporal regions. Supplementary Figs. S24 and S25 provide surface maps for the female 4-year and the male 2- and 4-year data. Supplementary file *test_metrics.csv* provides all metrics. Largest and smallest skewness and excess kurtosis also differed between male and female models for both timepoints. These results suggest that there may be sex differences in estimating ROI-wise C- or B-Norms.

ROI-wise comparisons of mean fits for the C-Norms and B-Norms reveal differential effects, that is, some ROI models explained more variance in the 2-year than in the 4-year follow-up or vice versa. For example, in the female longitudinal B-Norm model for a section of the posterior cingulate cortex (i.e., 31pv) of the right hemisphere, we found higher explained variance in the 2-year than in the 4-year follow-up data, whereas for the right PEF (premotor eye field) ROI the model explained more variance in the 4-year follow-up data. The full range of these results is available in the supplements (see supplementary Figs. S8, 9, 14, 15, 17, and 18).

To rule out that differences in C-Norm and B-Norm models may be solely attributed to age differences in the training set, we performed a supplemental analysis. Specifically, B-Norm models were trained using a broader age range, as they were trained using age at baseline and age at a follow-up timepoint, whereas the C-Norm models were only trained using baseline age. To investigate the degree to which such age differences may impact the model results, we trained another C-Norm model with the age at the 2-year follow-up (which we called C-Norm-2year) and a C-Norm model combining the baseline and 2-year follow-up data (termed C-Norm-bl+2year). This analysis revealed no age-range bias for our C-Norm models as performance measures and obtained deviation scores where highly similar to the C-Norm models trained using only baseline age (Supplementary Sect. Integrating Baseline Thickness Measures as Predictors for Norms in Longitudinal designs).

### Association with puberty scores

We investigated whether deviation scores of the 2-year or 4-year follow-up test data were associated with puberty stage as rated by the youths’ caregivers. Specifically, for each of the 360 ROIs, for each model and for each sex, we tested for linear associations, using generalized linear models (GLMs), between pubertal scores (PDS) and the deviations (z-scores) from B-Norm and C-Norm model, with Benjamini-Hochberg (BH) false discovery correction applied across tests.

**Fig. 3:**
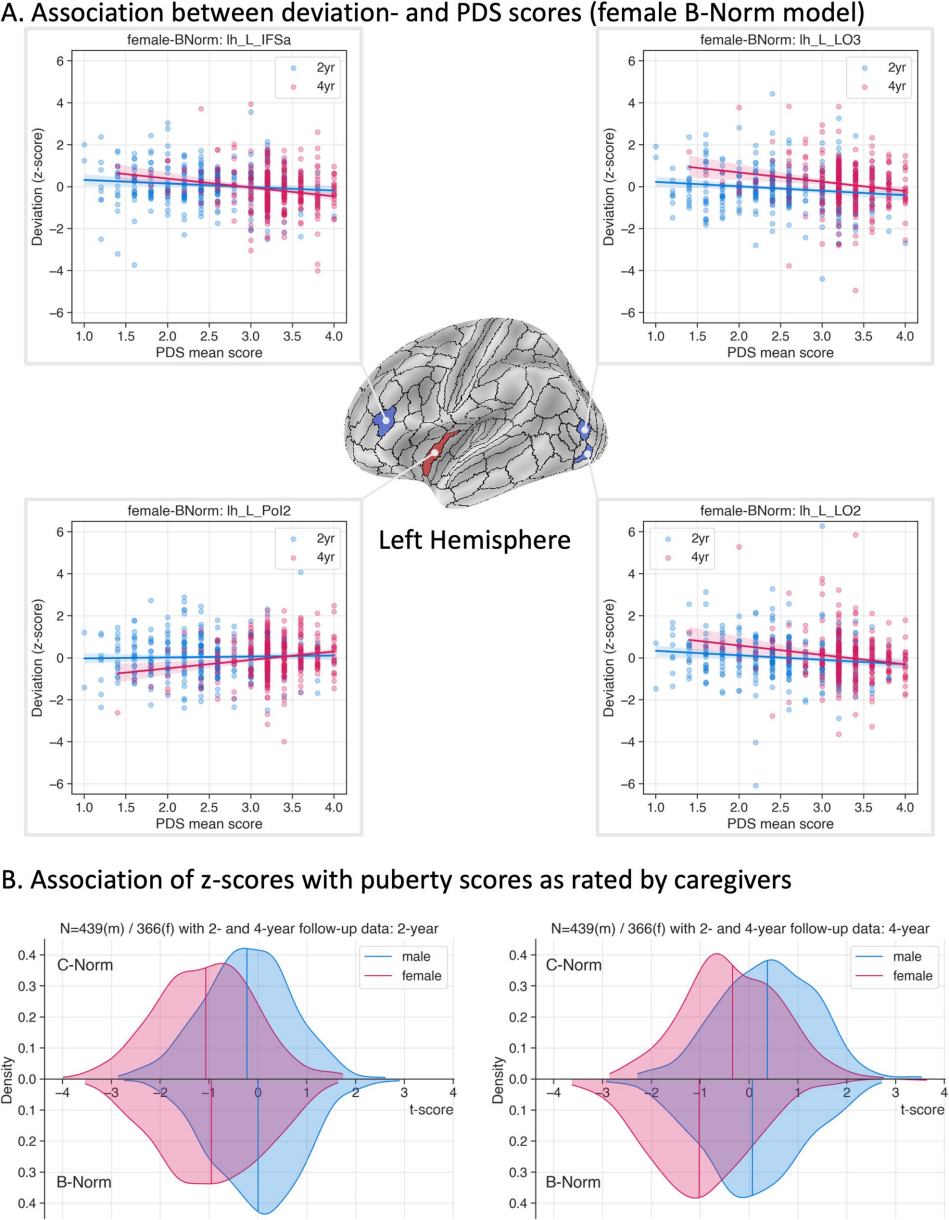
Association of deviation scores with puberty. Panel **A** shows a spatial surface map with the statistically significant areas for the female Baseline-Conditioned normative (B-Norm) models and the respective scatter plots and regression lines. The three areas marked in blue correspond to negative z-statistics as obtained from a GLM testing “PDS ~ z”, whereas the red marked area corresponds to the positive z-score. Blue (2-year) and red (4-year) colored dots within the scatter plots correspond to a single individual. Colored lines reflect the respective regression line of the data; patches around lines indicate the 95th confidence interval. The scatter plot for the significant female cross-sectional normative (C-Norm) model is available in the supplementary material (Fig. S26). Panel **B** shows the overall distributions of the GLM z-statistics for this analysis. Colors indicate sex (red = female, blue = male), curves above the x-axis correspond to the associations for the C-Norms, whereas those below correspond to B-Norms.

For C-Norms, only one association survived correction for multiple comparisons; specifically in 2-year follow-up data for females in the left hemisphere’s area 31pv, a part of the left posterior cingulate cortex (z=−3.985, *p* < 0.02442, BH corrected, *N* = 366, df_model_=1, df_resid_=364, scale = 0.41, deviance = 149.5). For female B-Norms, four areas of the left hemisphere survived correction; specifically for two subsections of the lateral occipital cortex (LO2: z=−3.597, *p* < 0.0362, BH corrected, df_model_=1, df_resid_=364, scale = 0.168, deviance = 61.15 and LO3: z=−3.540, *p* < 0.0362, BH corrected, df_model_=1, df_resid_=364, scale = 0.1682, deviance = 61.22), an area in the lateral frontal lobe (IFSa: z=−3.619, *p* < 0.0362, BH corrected, df_model_=1, df_resid_=364, scale = 0.168, deviance = 61.13), and an area of the insula (PoI2: z = 3.632, *p* < 0.0362, BH corrected, df_model_=1, df_resid_=364, scale = 0.168, deviance = 61.11). These results suggest, that with progression through puberty, areas LO2, LO3, and the IFSa exhibit decrease in cortical thickness, whereas parts of the insula appeared to show positive deviation. A corresponding surface map for the significant B-Norm areas is depicted in Fig. 3A (for the 31pv of C-Norms see supplementary fig. S26). Furthermore, the distributions of t-scores across ROIs in Fig. 3B suggest that there is an average negative shift for the females in the 2-year data for both the C-Norms and B-Norms. This negative shift for the females is lost for the C-Norms in the 4-year follow-up data but remains for the B-Norms, indicating that such models may better capture a relationship between puberty progression and changes in cortical thickness.

We also performed the same analysis using the simple difference between the 2- or 4-year data and baseline cortical thickness (i.e., 2-/4-year minus baseline). This is the most common approach for longitudinal data but yielded no significant associations. The ROI-wise statistics for this approach are available as supplementary csv files.

In addition, we adjusted our train- and test set for family structure since the ABCD dataset has high proportions of siblings/twins. For this approach, we found no difference in performance and predicted norms between the models trained using all subjects and those trained with data accounted for family structure (see Supplements Sect. Adjusting for family structure in training- and test-set). However, we did not find the previously reported statistically significant associations in the aforementioned brain areas. Significance values increased to *p* = 0.0965 for the left 31pv and to *p* = 0.0636 for the left LO2, IFSa, PoI2 and LO3. This could be because sample sizes decreased by 37 and 26 in the males (new *N* = 402) and females (new *N* = 340) respectively.

Lastly, we added BMI and socioeconomic status (SES) as covariates in our GLM analyses. That is, the formula for the GLM then was “PDS ~ z*BMI*SES” yielding main as well as interaction effects. Including these variables no longer yields significant associations between deviation and PDS scores. However, BMI was highly associated with PDS in nearly all brain regions for both males and females [56] as well as both follow-up timepoints, which poses issues regarding co-linearity. SES was also associated in nearly all brain areas, however only for both male and female CNorm-2year data, for female-Cnorm-2year data, and for both male and female BNorm-2year data. Full tabular statistical data files for these analyses are also available as supplementary files.

### Validation of normative models using puberty subgroups

**Fig. 4:**
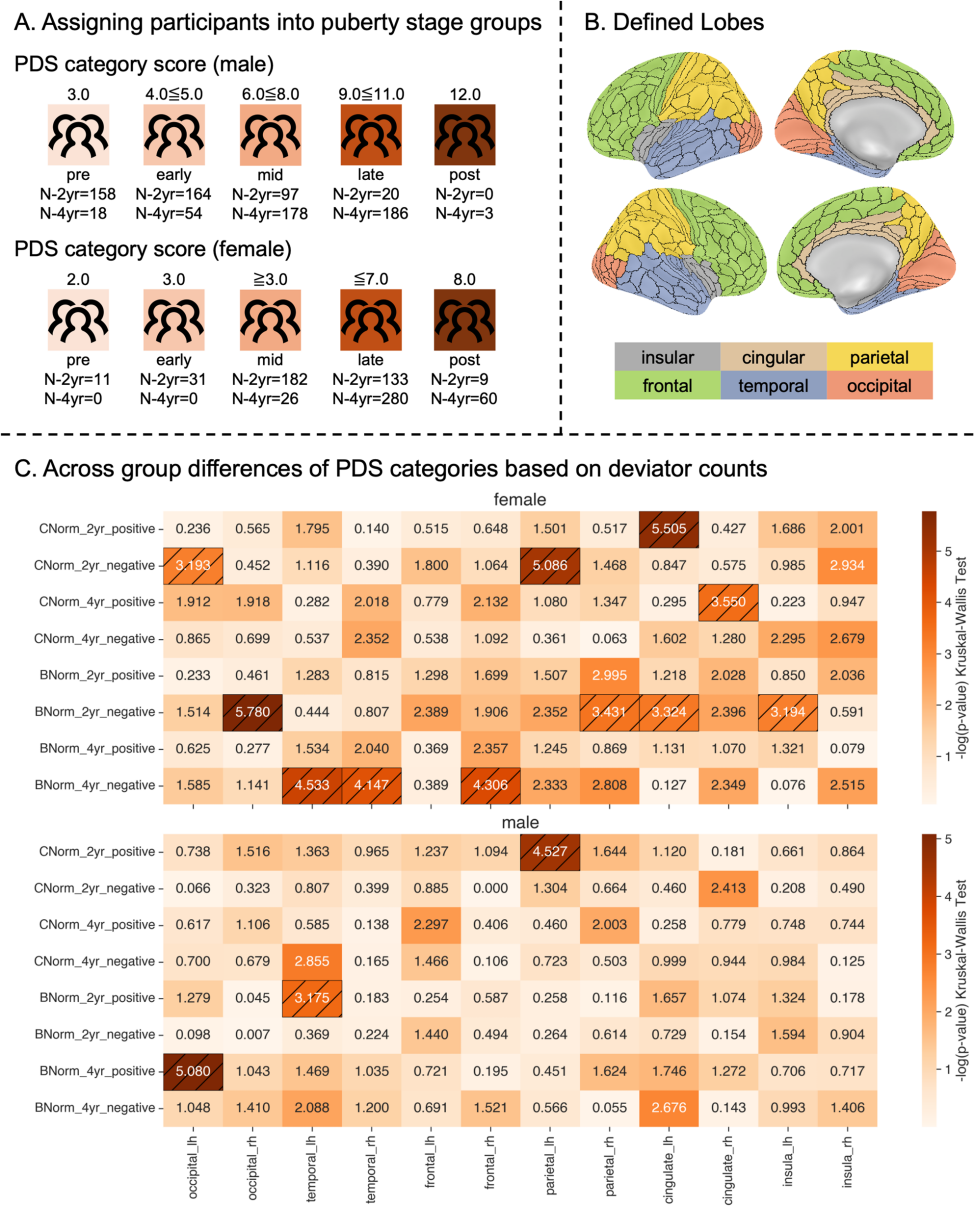
Definition and differences in pubertal stages for lobe-wise positive and negative deviation counts. Panel **A**) shows the definiton of pubertal stages/categories based on puberty scores (see Herting et al., and Kraft et al.). Panel **B**) illustrates which regions of the Glasser atlas were combined into lobar regions (definition based on [54]. Panel **C**) shows -log-transformed p-values according to Kruskal-Wallis omnibus test for differences in positive/negative deviation counts between pubertal categories (pre-, early-, mid-, late-, and post-pubertal) for both timepoints using the C-Norms or B-Norms for female (top) and male (bottom) data. Hatched cells indicate FDR corrected significance.

We further evaluated whether positive or negative deviation counts, defined as the sum of regions with deviation scores greater than or less than z = ±1.96, were associated with specific pubertal stages. To this end, we grouped participants according to their pubertal stage (pre, early, mid, late and post pubertal, Fig. 4A) and analyzed deviations at the level of lobes (Fig. 4B). For females (see Fig. 4C top), 11 Kruskal-Wallis omnibus tests yielded significant group effects. In cross-sectional normative models (C-Norms), marginally significant group differences were found among negative deviators at the 2-year follow-up in the left occipital (H(4) = 9.96, *p* = 0.041) and parietal (H(4) = 14.38, *p* = 0.0062) lobes; Dunn’s post-hoc tests suggested a mid- vs. late-pubertal group difference in the parietal lobe (*p* = 0.0053) but not in any of the pubertal stages for the occipital lobe (min *p* = 0.139 for mid- vs. late-pubertal). For positive deviators, we found significant effects in the left cingulate for the 2-year follow-up data (H(4) = 15.33, *p* = 0.0041), with early- vs. late- (*p* = 0.036) and mid- vs. late-pubertal (*p* = 0.0101) contrasts, and in the right cingulate for the 4-year follow-up (H(2) = 7.10, *p* = 0.0288), though pairwise comparisons were not significant (min *p* = 0.0705 for late- vs. post-pubertal).

B-Norms showed significant effects for negative deviators in the right occipital (H(4) = 15.95, *p* = 0.0031; mid- vs. late-pubertal *p* = 0.0031), right parietal (H(4) = 10.53, *p* = 0.0324; min pairwise *p* = 0.069 for early- vs. mid-pubertal), and the left cingulate (H(4) = 10.276, *p* < 0.0360, min *p* = 0.221 for mid- vs. late-pubertal) after two years. At 4-years follow-up, significant overall pubertal stage differences were found for negative deviators in the left (H(2) = 9.07, *p* = 0.0108) and right (H(2) = 8.30, *p* = 0.016) temporal lobes, and the right frontal lobe (H(2) = 8.61, *p* = 0.0135), all driven by mid- vs. late-pubertal contrasts (*p* = 0.095, *p* = 0.012, and *p* = 0.01, respectively). Corresponding p-values are visualized in Fig. 4C; significant cells are hatched in black. We used Bonferroni correction to adjust p-values for multiple comparisons for Dunn’s post-hoc tests. These results suggest, that for females, the largest differences between pubertal stages in relation to extreme deviations from the norm can be found between mid- and late pubertal participants.

For males (see Fig. 4C bottom), we only found three significant Kruskal-Wallis omnibus tests. That is, for the C-Norms, we found significant group differences only in the left parietal lobe of negative deviators in the 2-year follow-up data (H(3) = 11.176, *p* < 0.0109, specifically for early- vs. mid-pubertal *p* = 0.0134). For B-Norms, we found marginally significant effects for positive deviators in the left temporal lobe (H(3) = 8.213, *p* < 0.042, specifically for pre- vs. early pubertal *p* = 0.0481) in the 2-year follow-up data and slightly stronger results in the left occipital lobe (H(4) = 14.364, *p* < 0.0062, specifically for mid- vs. late-pubertal *p* = 0.038) for 4-year follow-up data.

## Discussion

Our study shows that Baseline-Conditioned Norms (B-Norms), which model future cortical thickness measures based on baseline cortical thickness, baseline age, and age at follow-up, appeared to capture more meaningful associations between brain structure and pubertal development than established Cross-Sectional Norms (C-Norms) in a longitudinal context of the ABCD study [39]. B-Norms revealed marginally significant region-specific deviation patterns associated with pubertal stage progression in females, particularly at later follow-ups, including four years after baseline. In contrast, these associations were absent or markedly weaker when using C-Norms. Despite being marginal effects, these results hint toward a benefit by priming models using baseline measures. Our perceived enhanced sensitivity of B-Norms likely stems from their ability to capture developmental changes occurring between baseline and follow-up, which are obscured when relying solely on age-based cross-sectional norms. Notably, the link between the number of negatively deviating regions within lobes and pubertal stage progression hints towards the potential of B-Norms to detect subtle, regionally specific brain changes during adolescence. However, these results need to be investigated further with more available data, e.g., using the next batch of the ABCD dataset.

Conversely, growth chart-style analyses did not reveal significant associations with pubertal status at this stage, suggesting that models using baseline cortical thickness to predict future thickness measures are potentially better suited for detecting nuanced personalized developmental trajectories.

Performance differences between the two model types indicated that B-Norms explained approximately 60% more variance in cortical thickness at both 2-year and 4-year follow-ups compared to C-Norms, which is to be expected given the design of our B-Norm models that use baseline measures of the variable of interest (i.e., cortical thickness). Supplementary analyses ruled out that differences in the age composition of data used in training of the models may have confounded predictions. Specifically, we found no evidence that an extended age range, e.g., combining the age range of baseline and the 2-year follow-up data, provided any performance benefit for the C-Norm models. In fact, we observed almost identical deviation scores for C-Norm models trained using only baseline age and those trained with combined baseline and follow-up age (see Fig. S7). Therefore, the major performance difference between models can be attributed to the use of longitudinal thickness data in modelling.

It should be noted that the C-Norm models in our analysis performed worse than what has been reported in prior work using similar models trained across the full lifespan [3]. It is likely that the narrow age range of the ABCD study sample accounts for the lower performance overall. However, this age range attributed performance drop cannot explain the performance difference of B-Norms and C-Norms in our work, given that the training data used for C-Norms and B-Norms is identical.

An important consideration of the comparison of both norms is that they capture different aspects of development and may therefore complement each other. C-Norms tell us how different someone at a given age is with respect to the population. B-Norms on the other hand tell us how stable someone is over time or development. We suggest using both models in tandem. For example, imagine a participant with an unusually large or small cortical thickness in a brain area at baseline and at follow-up timepoints. The C-Norm model will flag this participant as unusual in all timepoints. If this participant’s thickness values within this area do not change dramatically during development, the B-Norm model will not flag this participant as unusual in later timepoints (for an example of this effect see Supplementary Sect. Within subject differences of extreme z-scores). This effect may make the B-Norm models especially interesting in longitudinal contexts, as these models may better identify disease related changes.

Investigating the spatial distribution of performances across the brain surface allowed us to further investigate performance differences between C-Norms and B-Norms. While B-Norms achieved largest explained variances in the occipital (Glasser: left/right PIT, VMV1, and left MT) and temporal (Glasser: right PHT, PH, TP0J2 and left TP0J1, TE1m and STSda) regions with similar patterns across sexes, explained variance was lowest in parietal, insular and to some degree in frontal regions. Prior work linked parts of the occipital cortex to pubertal changes, including negative associations with testosterone in females [57]. Although a positive association was reported in males, the relatively early pubertal stage of ABCD male participants may explain the absence of this effect here. Interestingly, Wierenga et al. (2022) reported an interaction of cortical thickness with age in the left insula for males [58]. Here, our B-Norms fit insular regions less well in females, possibly reflecting more variability in females. Furthermore, we found inconsistent results in frontal and parietal regions for male and female C-Norms, areas previously tied to pubertal development [59]. This could be a potential reason for the lower predictive performances of C-Norms compared to B-Norms.

As was highlighted in a recent commentary, it is important to investigate higher-order moments of model derived deviation score distributions in addition to the mean fit to properly assess model performance [55]. Therefore, we also investigated differences in skewness and excess kurtosis for our C- and B-Norms. This analysis suggested that deviation scores obtained from C-Norm models were more normally distributed than for B-Norm models (excess kurtosis and skewness are closer to 0). The kurtosis results suggest that regional B-Norms are generally more sensitive to changes between visits, which can be seen by the narrower centiles of the normative plots as opposed to those of the C-Norms (see Fig. 3C).

While highest performance in some circumstances is desirable it is important to validate models using meaningful data. To this end, we examined whether deviation scores obtained from our models show a relationship with progression through puberty. The three regions that we found to be significantly associated all showed a negative association between pubertal process and deviation scores and were all in B-Norms of females. These negative associations with PDS may be consistent with previous findings in relation with estradiol levels [60]. Area left PoI2 in the insula was associated with positive deviations (i.e., an increase in cortical thickness) with progression through puberty (higher PDS). Overall, this analysis suggested that deviation scores as obtained by our B-Norms were slightly more negatively associated with pubertal development in females at the 4-year timepoint than the cross-sectional models. This can be seen by the slight leftward shift of the respective distribution as seen on the right side of Fig. 3B. These results suggest that while a better mean fit of the respective population technically means less variance in the residuals which often leads to fewer associations with phenotypic variables and may therefore not be useful in clinical settings. This issue has previously been reported for brain age prediction where models with very high fit can yield weaker associations with clinical variables (Bashyam et al.[61] and see Hahn et al. [62]). Despite this effect our B-Norms still pick up on unexpected or unusual (e.g., early or late puberty onset in relation to the baseline cortical thickness and age) changes between the baseline and 4-year follow-up. Additionally, such changes could be quite small and yet lead to large deviations. This may be due to the high excess kurtosis, which indicates narrow distributions where even small changes produce large deviation scores. That these small changes align with pubertal progress suggests that incorporating baseline measures (B-Norms) increases sensitivity. However, given the small sample size of our test set these results should be interpreted with caution at this stage. Applying our models to the next ABCD release could shed more light on them. This would be interesting as longer intervals between baseline and follow-up may amplify this effect by detecting more unusual changes, which should be tested in the future. In an auxiliary analysis we investigated whether family structure had an impact on this association analysis. After adjusting for family relationships in the training and testing set, the statistically significant associations mentioned before vanished. We argue that this could be due to the sample size drop in the test set since the performance and predicted norms of the models trained and evaluated using family-adjusted data did not differ from those obtained from models trained on all participants (see Supplements Sect. Adjusting for family structure in training- and test-set).

Similar to previous studies that applied normative modelling to clinical populations, we investigated whether aggregated deviations from normative brain development, summed across regions of interest (similar to total outlier count, see Verdi et al. [27]; Wolfers et al. [17]), were sensitive to distinct pubertal stages. We observed sex-specific differences in how deviation counts varied across pubertal stages. Female participants showed greater effects: four lobes exhibited significant differences across stages in the C-Norms, and seven in the B-Norms, compared to only one and two lobes respectively in males. Pairwise comparisons in the female 2-year follow-up cohort revealed that deviation scores differed significantly between mid- and late-pubertal stages in the right occipital and left cingulate lobes, and between early- and mid-pubertal stages in the right parietal lobe. Interestingly, in the 4-year follow-up data, negative deviations, which reflect cortical thinning relative to normative expectations, were observed in the bilateral temporal and right frontal lobes, primarily distinguishing mid- from late-pubertal females. These lobes are known to undergo significant cortical thinning during adolescence [63, 64]. Thus, our findings are in line with previously established findings that this process is stage specific, with late pubertal stages marked by increased deviation from normative developmental trajectories given a certain baseline and age. These findings align with previous literature emphasizing more dynamic neurodevelopmental trajectories in females during puberty, possibly due to earlier onset and more rapid progression of hormonal changes.

Reduced cortical thickness during puberty has been previously linked to synaptic pruning, myelination, and hormonal influences [65, 66]. In particular, thinning in temporal and frontal areas has been associated with cognitive maturation and may reflect the refinement of higher-order processes such as emotion regulation and social cognition. Such cognitive functions are especially sensitive to pubertal timing [67, 68].

In a final validation step, using our normative models related to growth charts used in pediatrics, we categorized participants according to percentile shifts into negative, stable and positive deviators by computing the difference between the deviation scores (i.e., z-scores) between the 2- and 4-year follow-up data. This analysis did not reveal any significant effects after correcting for multiple comparisons rendering these results difficult to interpret (see Supplements Sect. Validation of normative models using percentile shifts). We believe that one reason may be our chosen zDiff threshold – which is one of many possible thresholds – and the still relatively small sample size of our test set. Without correcting for multiple comparisons, we found marginal differences in associated regions between females and males (see Figs. S28 and 29). Interestingly, if these results were to hold, ROI-wise male C-Norm and B-Norm models could show stronger and more differences than female models in this percentile shift analysis. This direction should be investigated further with future ABCD releases.

## Limitations

A limitation of this study, much like for any other current development of longitudinal methods, is restricted by the availability of large-scale datasets covering the age-range of interest. The degree to which our results translate beyond the population characteristics of the ABCD study cohort [39] remains to be investigated with future releases of new longitudinal datasets. Fortunately, such models can easily be extended, adapted, or retrained with new data releases.

Sample characteristics also apply when interpreting our results on sex differences. The majority of the male participants of the ABCD dataset did not yet fully progress through puberty, which likely explains the lack of puberty related associations with deviation scores. We argue that this has no implications for the results of the female models, as we have trained sex specific models and because it has been shown previously that brain development during puberty is different for males and females [58]. A further limitation is that adding BMI and SES as confounds for our statistical analyses yields non-significant results. However, we are not the first to report a loss of effects incorporating these variables [41].

It should also be considered that at this point we cannot for certain attribute the loss of significant results in the association analysis when we accounted for familial relationship to either family-relationships or the drop in sample size. This should be investigated with future ABCD releases.

Another limitation of the current study is the sole focus on puberty related changes. While interesting in their own rights, validation of C- and B-Norms using clinical variables (e.g., depression rating scales or similar) is necessary to estimate their utility in, for example, detecting and predicting developmental or age-related disease trajectories. Thus, future studies should incorporate clinical values and extended our proposed models. Furthermore, interpretation of the tested B-Norm models may be difficult due to their complexity. Given the required baseline features, cortical thickness and age as well as the age at a future timepoint, the model trajectories and centiles are estimated based on variable baseline cortical thickness depending on the baseline and future age. In theory this means that for each baseline and follow-up age per region of interest a growth chart must be generated (see Supplementary Fig. S23 for two additional examples).

## Conclusion

In this study, we present a developmental application of normative modeling and demonstrate that Baseline-Conditioned Norms (B-Norms) utilizing longitudinal data yield deviation scores that are meaningfully associated with pubertal development. These associations were more pronounced in females than in males, likely reflecting the greater puberty-related variance in females in the respective age period. While still at an early stage, this Baseline-Conditioned modeling framework may serve as a valuable complement to established cross-sectional norms, and be of particular value in the study of disease associated longitudinal brain change. Our findings further underscore the potential of this modeling approach to capture individual variability in different developmental phases.

## Supplementary Information

Supplementary Material 1.

Supplementary Material 2.

Supplementary Material 3.

## Data Availability

The datasets generated and analyzed during the current study are available via dedicated data use agreements with the Adolescent Brain Cognitive Development Study^®^ (ABCD, [https://abcdstudy.org]. Code is available on P.S. github-repository: [https://github.com/PhilippS893/b-norm_modelling].

## Abbreviations

ABCD: Adolescent Brain Cognitive Development
B-Norm(s): Baseline-Conditioned Norms
C-Norm(s): Cross-Sectional Norms
PDS: Pubertal Development Score
SD: Standard deviation
